# Progress in the Study of Animal Models of Metabolic Dysfunction-Associated Steatotic Liver Disease

**DOI:** 10.3390/nu16183120

**Published:** 2024-09-15

**Authors:** Yu Fu, Yuxin Hua, Naqash Alam, Enqi Liu

**Affiliations:** 1 MOE Key Laboratory of Cell Activities and Stress Adaptations, School of Life Sciences, Lanzhou University, Lanzhou 730000, China; fuy2017@lzu.edu.cn (Y.F.); 220220932951@lzu.edu.cn (Y.H.); 2 Laboratory Animal Center, Xi’an Jiaotong University Health Science Center, 76 Yanta West Road, Xi’an 710061, China; naqashalam@stu.xjtu.edu.cn

**Keywords:** MASLD, animal model, physiology, pathology

## Abstract

Metabolic dysfunction-associated steatotic liver disease (MASLD) has recently been proposed as an alternative term to NAFLD. MASLD is a globally recognized chronic liver disease that poses significant health concerns and is frequently associated with obesity, insulin resistance, and hyperlipidemia. To better understand its pathogenesis and to develop effective treatments, it is essential to establish suitable animal models. Therefore, attempts have been made to establish modelling approaches that are highly similar to human diet, physiology, and pathology to better replicate disease progression. Here, we reviewed the pathogenesis of MASLD disease and summarised the used animal models of MASLD in the last 7 years through the PubMed database. In addition, we have summarised the commonly used animal models of MASLD and describe the advantages and disadvantages of various models of MASLD induction, including genetic models, diet, and chemically induced models, to provide directions for research on the pathogenesis and treatment of MASLD.

## 1. Introduction

Non-alcoholic fatty liver disease (NAFLD) is the leading cause of liver disease in most countries around the world [1]. The global prevalence of NAFLD is gradually increasing and currently estimated to be over 30% [2]. However, this prevalence varies worldwide due to differences in demographic characteristics and geographic epidemiology. The highest prevalence is in the Middle East and South America, and the lowest in Africa [3]. However, in Asian countries, most NAFLD patients are not obese and have lower body mass indexes, while NAFLD patients in Western countries tend to be more obese and insulin resistant [4]. In the United States, the incidence of NAFLD is expected to increase significantly, increasing by 21% from 83.1 million cases in 2015 to 100.9 million in 2030. Furthermore, the prevalence of NASH has increased by 63%, accompanied by a significant increase in associated mortality rates [1]. Due to the higher prevalence and mortality of NAFLD, there is significant interest in investigating its underlying pathophysiology to identify potential therapeutic targets.

**Figure 1:**
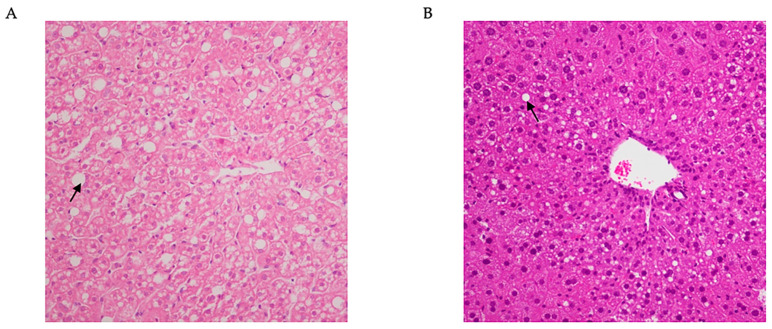
Histological lesions of hepatic steatosis in nonhuman primates and ApoE^-/-^ mice. Panels (**A**,**B**) show hematoxylin and eosin (HE) staining results of fatty liver induced by a high-fat diet in nonhuman primates and ApoE^-/-^ mice, respectively. The arrow points to the histological lesion of typical liver steatosis with vesicular fat accumulation. Data were obtained from the results of experiments performed in our laboratory (unpublished data).

In 2023, the Liver Association proposed replacing the term NAFLD with metabolic dysfunction-associated steatotic liver disease (MASLD) and NASH with metabolic dysfunction-associated steatohepatitis (MASH) [5]. The new nomenclature better reflects the pathophysiological significance of liver disease [6,7]. The hallmark of MASLD is the accumulation of lipids in the hepatocytes leading to steatosis (Figure 1). Patients with MASLD frequently exhibit metabolic conditions such as obesity, insulin resistance, or type 2 diabetes [3]. The increase in intrahepatic lipids drives the progression of simple steatosis into MASH [8]. The histopathological features of MASH include hepatocellular steatosis, hepatic lobular ballooning, inflammation, and fibrosis, making it a necrotizing inflammatory disease [9].

**Figure 2:**
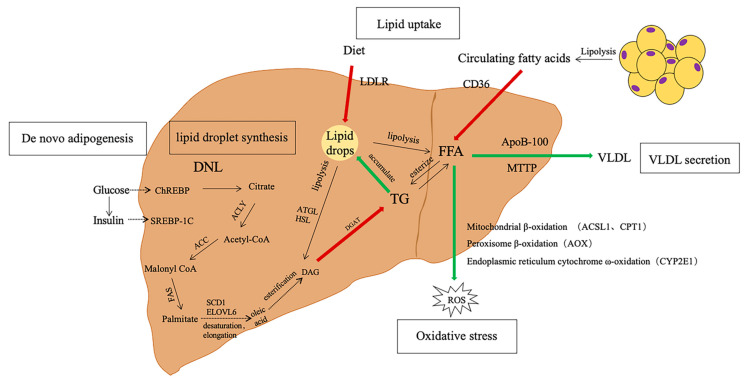
Hepatic free fatty acid (FFA) synthesis, metabolic processes, and related major genes. Fatty liver is formed mainly by an imbalance between the increase in fatty acids (red arrows) and their metabolism (green arrows). Glucose and insulin activate the transcription factor sterol regulatory binding protein (SREBP-1C) and the transcription factor carbohydrate response element binding protein (ChREBP), respectively, to synthesize triglycerides (TG) and produce FFA. Hepatic FFAs can also be derived from the diet and circulating fatty acids. In the liver, FFAs are stored as triglycerides (TG) to form lipid droplets or exported as very low-density lipoproteins (VLDL). Excess FFAs undergo beta-oxidation in mitochondria or degradation in lysosomes for reuse. FFA, free fatty acid; DNL, De novo lipogenesis; ChREBP, carbohydrate response element binding protein; SREBP-1C, sterol regulatory element binding protein 1-C; ACC, acetyl coenzyme A carboxylase; FAS, fatty acid synthase; SCD1, stearoyl coenzyme A desaturase 1; ELOVL, extra-long-chain fatty acid extension; DAG, diacyl triglyceride; DGAT, diester acylglycerol acyltransferase; HSL, hormone-sensitive lipase; ATGL, adipose triglyceride lipase; CD36, lipid transporter protein; LDLR, low density lipoprotein receptor; ACC, acetyl coenzyme A carboxylase; FFA, free fatty acid; TG, triglyceride; VLDL, very-low-density lipoprotein; MTTP, microsomal triglyceride transfer protein; ApoB100, apolipoprotein B100; ROS, reactive oxygen species; ACSL1, long-chain acyl coenzyme A synthase 1; CPT1, carnitine palmitoyltransferase 1; AOX, fatty acyl coenzyme A oxidase; CYP2E1, cytochrome P4502E1.

The main cause of the formation of hepatic steatosis is the imbalance in the uptake, synthesis, and metabolism of lipids by the liver, which ultimately affects the accumulation of lipid droplets in hepatocytes. The fundamental characteristic of hepatic steatosis is the accumulation of at least 5% fat in the hepatocytes. This condition is characterized by the formation of lipid droplets in the liver due to the excessive accumulation of triglycerides. These droplets are surrounded by a phospholipid monolayer that contains cholesterol [10]. When insulin resistance occurs in the body, the liver absorbs excess glucose and insulin [11]. This process activates the transcription factor carbohydrate response element-binding protein (ChREBP) and the transcription factor sterol regulatory element-binding protein 1c (SREBP-1c), leading to the increased production of free fatty acids (FFAs) by the liver [12]. Hepatic uptake of circulating fatty acids is mediated by the fatty acid transport protein (CD36) [13], which in turn affects FFA uptake. At the same time, dietary triglycerides (TG) are hydrolyzed by pancreatic lipase and then emulsified by bile acids in the intestine to synthesize new celiac particles in the blood circulation. After that, most of the TG in the celiac particles are hydrolyzed by lipase catalysis to form celiac residues, mainly from cholesterol esters, which are finally recognized by low-density lipoprotein receptor (LDLR)-mediated endocytosis into the liver and further fused with lysosomes, and cholesterol esters are broken down into cholesterol and fatty acids [14]. De novo lipogenesis (DNL) allows the synthesis of new FFA from citrate. Initially, citrate is catalyzed by ATP-citrate lyase (ACLY) to produce acetyl coenzyme A, which is synthesized into malonyl coenzyme A by acetyl coenzyme A carboxylase (ACC), and then malonyl coenzyme A is converted to palmitate by fatty acid synthase (FAS). After the desaturation, elongation, and esterification steps, the liver finally stores FFA in lipid droplets as TG or secretes it outside the liver as very low-density lipoprotein (VLDL) through the combined action of MTTP and ApoB-100, resulting in excess FFA liver mitochondrial dysfunction, cytochrome production and endoplasmic reticulum stress, and reactive oxygen species (ROS) production. Among them, long-chain acyl coenzyme A synthase 1 (ACSL1) and carnitine palmitoyltransferase 1 (CPT1) on the outer mitochondrial membrane induce β-oxidation in a complementary manner. In contrast, fatty acyl-coenzyme oxidase (AOX) and cytochrome P4502E1 (CYP2E1) mediate peroxisome β-oxidation and ω-oxidation, respectively. Meanwhile, lipid droplets accumulated in the liver are ultimately hydrolyzed back into glycerin and FFA and reused by the body, mainly through adipose triglyceride lipase (ATGL) and hormone-sensitive lipase (HSL). The uptake, synthesis and metabolism of FFAs in the liver as well as the key genes involved in these processes are shown in Figure 2. (i) Dietary exogenous fatty acid and white fat uptake are used to synthesize FFA; (ii) synthesis of FFA from de novo fatty acids; (iii) FFA output by lipid oxidation and very low-density lipoproteins; (iv) excessive accumulation of triglycerides in the liver to form lipid droplets.

The pathogenesis of MASH was originally explained by the “two-hit” hypothesis: the “first hit” involves lipid accumulation, fatty acid oxidation, and reduced VLDL secretion in the liver, while the “second hit” involves mitochondrial dysfunction and inflammation caused by steatosis and ultimately leads to MASH [15]. MASH can further develop into cirrhosis and even liver cancer [16]. However, the “two-hits” theory does not consider the various factors that influence disease progression, such as genetics and metabolism. In contrast to the “two-hits” theory, the “multi-parallel hypothesis” theory considers the role of various factors in the disease progression and is consistent with the pathological process of MASH. MASH is the result of a combination of factors including genetic factors, gut microbiota, insulin resistance, oxidative stress, adipocytokines, cytokines, etc., which may play an important role in the development and progression of liver fibrosis and inflammation [17,18]. However, a further investigation of the pathogenesis of MASLD in humans requires more appropriate disease models that can mimic the process of disease onset and progression. Currently, various models for the pathogenesis of MASH have been developed, including genetic models [ob/ob mice], diet-induced [high-fat diet, methionine and choline-deficient diet (MCD)], fructose-induced diet, and chemical-induced injury [19]. Although all of these models exhibit some of the same histological changes that are characteristic of MASLD in humans, there are uncertainties as to whether they reflect the pathogenesis of human diseases. For example, leptin deficiency in ob/ob mice led to an increase in food intake and body weight, in addition to impaired insulin secretion [20]. However, after treatment with leptin, ob/ob mice showed not only a reduction in abnormal indicators, but also a partial improvement in liver steatosis [20]. In contrast, mice on a high-fat diet show increased body fat, elevated triglycerides, and higher insulin levels, which correlate with the fat content of the diet [21]. However, the relatively long duration of pathological processes induced by the high-fat diet has drawn more attention to dietary models that can rapidly mimic MASLD and MASH. The new dietary models typically contain high levels of fructose or sucrose, a high fat content, and elevated cholesterol. Diets containing high fructose, sucrose, and high fructose corn syrup have been shown to induce fatty liver in experimental animals and also influence TG elevation and effects on insulin resistance [22,23,24,25,26]. Therefore, different experimental animal models and induction methods may exhibit different results, which means that a detailed comparison of each model and modeling method is required.

This paper examines the characteristics of rodent and non-primate models and summarizes commonly used models for inducing MASH, including genetic, diet-induced, and chemical injury models. It compares these models based on their evolution over time and their characteristics, correlating them with the progression of human MASH to advance research on its pathogenesis.

**Figure 3:**
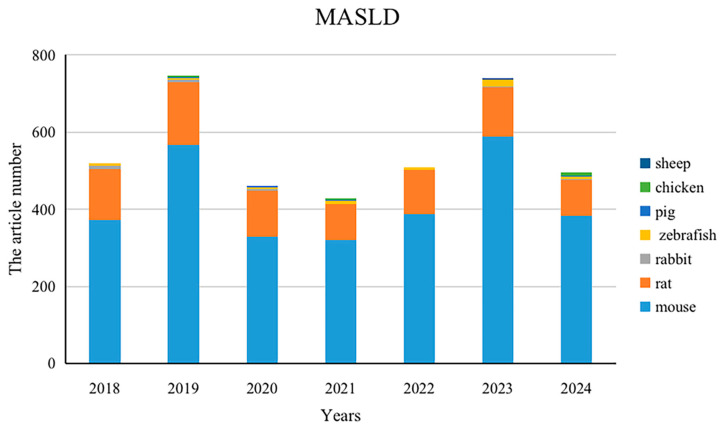
Number of animal models used in the last seven years. There were 519 articles in 2018, 744 articles in 2019, 461 articles in 2020, 425 articles in 2021, 508 articles in 2022, 737 articles in 2023, and 491 articles in 2024. Among the 3885 articles, there were 2949 mouse models, 842 rat models, 52 zebrafish models, 23 rabbit models, 13 pig models, five chicken models, and one sheep model.

**Figure 4:**
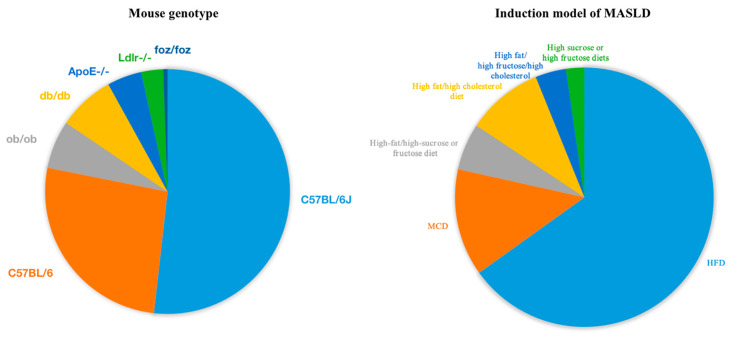
Summary of mouse gene knockout models and MASLD induction methods. The C57BL/6J and C57BL/6 mouse background is predominantly used, including knockout models such as ob/ob, ApoE^-/-^, Ldlr^-/-^, db/db, and foz/foz. For MASLD induction, HFD is most commonly used, followed by the MCD diet, high fat/high cholesterol diet, high-fat/high-sucrose or fructose diet, high fat/high fructose/high cholesterol, high sucrose or high fructose diets.

We searched the PubMed database using the keyword “non-alcoholic fatty liver disease”, because a systematic search using the new term “MASLD” could not fully locate previous studies. A total of 24,737 articles, including 3885 animal studies, were retrieved over the past 7 years (2018–2024), using animal models including mouse, rat, zebrafish, pig, rabbit, chicken, and sheep (Figure 3). Among the most commonly used mouse models, the C57BL/6J and C57BL/6 background mouse models are widely used for MASLD studies, and common knockout genotypes include ob/ob, ApoE^-/-^, Ldlr^-/-^, db/db, and foz/foz. The high-fat diet (HFD) is the most common induction method, followed by MCD (Figure 4).

## 2. Commonly Used Rodent and Non-Human Primate Models

### 2.1. Rodents

The selection of appropriate animal models is crucial for elucidating the pathophysiological mechanisms underlying MASLD. The most commonly used rodent models are mice and rats. C57BL/6 mice exhibit reduced hepatic steatosis and lower triglyceride levels; however, they show more pronounced inflammatory damage and increased lipid peroxidation [27]. However, in the case of MASH induced by various diets in rats, males demonstrated more pronounced steatosis, greater lipid deposition, and more extensive liver injury compared to females [28]. As a result, the degree of liver steatosis and injury varied significantly in models induced in different species and sexes; mouse and rat genetic mutation models showed different characteristics in MASLD studies (Table 1).

Leptin is a key regulator of lipid storage and regulates food intake via the hypothalamus, and both mouse and rat models exploring leptin dysfunction exist [29,30,31,32,33]. For genetic mouse models, there are ob/ob mice and db/db mice both affected by genetic mutations and receptor gene mutations for leptin function, respectively [29]. The ob/ob mice themselves are extremely obese and exhibit severe insulin resistance, hyperinsulinemia, and hyperglycemia as metabolic features [30]. The obesity profile of db/db mice is very similar to that of ob/ob mice, and due to their own unstable expression of leptin levels, they develop more severe diabetes than ob/ob mice, but have less hepatic steatosis than ob/ob mice [29]. In genetic rat models, such as leptin-receptor-deficient fa/fa rats, gene deletion leads to obesity, hyperlipidemia, and impaired insulin metabolism [34]. Although fa/fa rats show reduced thermogenic response and oxygen consumption, they do not show abnormalities in total energy expenditure [32]. The association between MASLD and atherosclerotic disease is controversial, as both are manifestations of metabolic syndrome. Pathological features of one disease can be influenced when they are induced in models of another disease [33,35]; therefore, the causal relationship between the two needs to be explored in depth. In genetic animal models, apolipoprotein E-deficient (ApoE^-/-^) mice and low-density lipoprotein-receptor-deficient (Ldlr^-/-^) mice are both well-established for studying atherosclerosis, and are also used with a high-fat diet (HFD) to simultaneously induce obesity, liver fibrosis, and atherosclerotic disease [36,37,38,39,40].

Therefore, for common genetic models of MASLD, researchers believe that genetic mutations alone are insufficient to develop MASH. Exogenous interventions (e.g., carbon tetrachloride or lipopolysaccharide) or dietary factors (e.g., high-fat diet or MCD diet) are required to further induce lesions [41,42]. Therefore, genetic models alone can only be used as supplementary factors to exacerbate the lesions.

**Table 1:** Commonly used transgenic animal models of MASLD rodents.

Genus	Models	Gene Description	Features	References
mouse	ob/ob	Leptin deficiency	Obesity, insulin resistance, steatosis	[43]
	db/db	Leptin receptor deficiency	Obesity, insulin resistance, steatosis	[44]
	foz/foz	Deficient in the Alstrom syndrome 1 gene	Obesity, steatosis, insulin resistance, high cholesterol levels	[45]
	LIR^-/-^	Liver-specific insulin receptor knockout	Hyperglycemia, hyperinsulinemia, high cholesterol levels	[46]
	LDLR^-/-^	LDL receptor-deficient	High cholesterol levels	[47,48]
	ApoE^-/-^	Apolipoprotein E deficient	High cholesterol levels	[49,50,51]
	FXR^-/-^	Farnesoid X receptor deficient	Decreased lipid and cholesterol metabolism	[52]
	CD36^-/-^	Fatty acid translocase CD36 deficient	High plasma fatty acid and triglyceride levels	[53]
	SREBP1c^-/-^	Sterol regulatory element-binding protein-1c deficient	Steatosis, insulin resistance, high triglyceride levels	[54]
	PPARa^-/-^	Peroxisome proliferator-activated receptor deficient	Severe fatty liver	[55]
	IL6^-/-^	Interleukin-6 deficient	Obesity, steatosis, insulin resistance	[56]
rat	fa/fa	Zucker obese rat	Obesity, insulin resistance, hyperglycemia	[34]

### 2.2. Non-Human Primates

Non-human primates are extensively used in MASLD research due to their close genetic and physiological similarities to humans. Commonly used non-human primates include rhesus monkeys and crab-eating monkeys. In contrast to rodent models, non-human primates have the advantage of spontaneously developing obesity with physiological and biochemical markers very similar to those in humans, such as body mass index, abdominal fat accumulation, insulin resistance, dyslipidemia, and diabetes mellitus. Genome-wide assays of rhesus monkeys have shown that the average sequence homology between humans and rhesus monkeys is approximately 93% [57]. Rhesus monkeys can spontaneously develop metabolic dysregulation associated with obesity. Analysis of the plasma lipidome of these monkeys revealed increased levels of free fatty acids (FFA) as well as the fatty acids palmitoleic acid and arachidonic acid [58]. Nagarajan et al. examined older bonnet monkeys and rhesus monkeys, revealing that the bonnet monkeys exhibited more severe steatosis compared to the rhesus monkeys. Additionally, the bonnet monkeys demonstrated associated hepatic portal fibrosis lesions [59]. In large-scale studies of rhesus monkeys, 35 out of 408 were found to exhibit susceptibility to metabolic syndrome over 18 months of monitoring [60]. Later, Zheng et al. conducted a more in-depth study on aged rhesus monkeys and found that MASLD can develop spontaneously with aging. This condition is accompanied by metabolic syndromes such as obesity and insulin resistance [61].

Therefore, for experimental animal models of MASLD, non-human primates are more consistent with the investigation of physio-pathological mechanisms in humans. Due to the highly similar pathologies of non-human primates and humans, the ultimate goal of the research is to be able to treat or prevent the occurrence of disease. In a recent study, Qu et al. used DT-109 (a glycine-based tripeptide) to treat MASH in crab-eating monkeys. This research elucidated the pathogenesis of drug-targeted MASH by focusing on the induction of fatty acid degradation and the enhancement of de novo glutathione synthesis to reduce both MASH and fibrosis [62]. This result substantially improves the translatability of MASH drug development up to the preclinical stage.

## 3. Common Diet-Induced Models of MASLD

The main MASLD diet induction models commonly used in experimental animals include a high-fat diet, methionine and choline-deficient (MCD) diets, choline-deficient L-amino acid (CDAA) diets, and high-sugar (fructose/sucrose) diets (Table 2). These diets are usually used alone or in combinations of several diets to induce steatosis and MASH [63,64,65].

### 3.1. High-Fat Diet

High-fat diet (HFD)-fed experimental animal models can effectively mimic the etiology of MASLD in humans. The HFD diet primarily allows experimental animals to consume energy from approximately 60% fat (within the range of 45% to 75%) compared to less than 20% energy intake in the regular diet [66]. Previous studies have shown that increased dietary fat content in mice [67] and rats [68,69] leads to insulin resistance and obesity. Different diet-induced obesity mouse strains revealed that C57BL/6J showed stronger glucose intolerance and insulin resistance than A/J mice, revealing that C57BL/6J is more consistent with studies related to human metabolic syndrome [67,70,71], which is closely associated with obesity and insulin resistance. Similarly, HFD induces hepatic steatosis and metabolic syndrome in crab-eating monkeys [72,73]. Although HFD mimics the MASLD steatosis process, the prolonged induction of high fat results was associated with only minor or absent liver fibrosis and inflammation-associated molecular markers [74]. Therefore, although HFD induction is useful for studying early-stage MASLD, it may be less effective for analyzing the detailed histological features of MASH lesions.

### 3.2. Methionine- and Choline-Deficient (MCD) Diet

The MCD diet is deficient in methionine and choline, both essential donors for lipid metabolism. A deficiency of these nutrients disrupts phospholipid synthesis, impairs lipoprotein secretion, and leads to mitochondrial dysfunction [75]. The MCD diet model induces rapid disease progression, with significant liver steatosis, inflammation, and fibrosis observed within 2 to 4 weeks of induction. However, mice on this diet tend to lose weight, exhibit markedly reduced fat distribution, and develop significant liver fibrosis without showing insulin resistance [76,77]. In contrast, studies on MCD-induced rats found that, although the liver exhibited more severe steatosis, there were no significant changes in inflammation or fibrotic lesions compared to the controls [78]. However, the MCD-induced rats also lost weight as mice [79]. In a study where both mice and rats were fed the MCD diet, the rats developed a more pronounced hepatic steatosis but exhibited less necroinflammation and no hepatic fibrosis. In contrast, mice showed a different pattern with significant necroinflammation and liver fibrosis [27]. Thus, while the MCD diet primarily affects fatty acid uptake and reduces VLDL secretion, leading to lipid accumulation in the liver and mimicking the classic histopathology of MASH, the same trends in obesity and insulin resistance are not typically associated with MASLD in humans [19]. The absence of these metabolic syndromes and the lack of a directly comparable diet in humans limit the applicability of the MCD diet model for studying human MASLD.

### 3.3. Choline-Deficient L-Amino Acid (CDAA) Diet

Similar to the MCD diet, the CDAA diet is deficient in choline, except that its protein is replaced by a mixture of L-amino acids [80]. The limitations of the MCD diet lead to weight loss, which can be mitigated by the CDAA diet, but as with MCD there are no metabolic features observed for any human MASLD [81]. Therefore, MCD and CDAA are only suitable for exploring the phenotypes of cirrhosis and hepatocellular carcinoma with intense fibrosis.

### 3.4. High Fat/Cholesterol Diet

Upon comparing the effects of dietary fat and cholesterol on MASH development, it was found that administering either fat or cholesterol alone increased hepatic fat deposition. However, more severe hepatic steatosis, inflammation, and fibrosis were observed when both fat and cholesterol were included in the diet [82]. Additionally, this diet was effective in inducing the hypercholesterolemic and obese features of MASH in humans [64].

### 3.5. High-Fat/Fructose Diet

Fructose is a dietary monosaccharide and one of the two most commonly used sweetening additives, where sucrose is a disaccharide composed of fructose and glucose, and high fructose corn syrup is a mixture of fructose and glucose monosaccharides [83]. Recent animal models of MASLD have predominantly focused on fructose, which is often used in combination with high levels of fat and cholesterol. This approach tends to be more effective for promoting hepatic steatosis and fibrogenesis and more similar to the pathogenesis of MASLD in humans [84]. Fructose can be used as a substrate for hepatic DNL, inducing steatosis and insulin resistance. Additionally, it inhibits fatty acid oxidation and contributes to endoplasmic reticulum stress [85]. Tetri et al. used a novel approach to induce MASLD by combining high fructose corn syrup with trans fats in a high-fat diet fed to mice. The high fructose promoted insulin sensitivity and obesity, while trans fats exacerbated hepatic steatosis and were associated with necroinflammatory changes [86]. Subsequent studies confirmed that the use of the same diet resulted in significant hepatic steatosis, dyslipidemia, and hepatic fibrosis. Furthermore, the MASH disease progression induced by this diet closely resembles the disease progression in humans [87,88]. However, considering that fibrosis is a critical marker of disease severity in MASH patients, Kohli et al. developed a rodent model incorporating a high-calorie diet enriched with medium-chain saturated fatty acids and high-fructose drinking water. This model was designed to more precisely replicate the obesity and insulin resistance characteristic of human MASH [89]. The role of high fructose diet in necroinflammation and fibrosis in MASH was further confirmed in a subsequent induction model in rats [90]. In addition to rodent studies, fructose has also been used in primate studies. In a study of crab-eating monkeys, a high-fructose diet significantly increased both hepatic steatosis and fibrosis [91]. Leigh Goedeke et al. also used high-fat, high-fructose diets to feed crab-eating monkeys and rhesus monkeys, and similarly found biochemical and histopathological features of MASLD [92]. In addition, a high fructose diet affects insulin resistance levels in rhesus monkeys in the short term and triggers metabolic syndrome [93].

**Table 2:** Characteristics of commonly used MASLD diet-induced models in experimental animals.

Models	Mechanisms	Obesity	Insulin Resistance	Inflammation	Liver Fibrosis	Duration of MASLD Induction	References
HFD	Metabolic abnormalities, oxidative stress and inflammation, insulin resistance	Y	Y	Y	N	8–24 weeks	[67,68,69,70,71]
MCD	Metabolic abnormalities, oxidative stress and inflammation, fibrosis	N	N	Y	Y	4–9 weeks	[76,78,94]
CDAA	Metabolic abnormalities, oxidative stress and inflammation, fibrosis	Y	N	Y	Y	4–9 weeks	[81,95]
HFD + Fructose	Metabolic abnormalities, oxidative stress and inflammation, mild fibrosis, insulin resistance	Y	Y	Y	Y	12–16 weeks	[86,87,88,89,90]
HFD + Cholesterol	Metabolic abnormalities, oxidative stress and inflammation, insulin resistance	Y	Y	Y	N	12–16 weeks	[96]
HFD + Fructose + Cholesterol	Metabolic abnormalities, oxidative stress and inflammation, fibrosis, insulin resistance	Y	Y	Y	Y	12–16 weeks	[82,97]

Note. Y. yes, N. no.

## 4. Chemical Substance Induction Model

### 4.1. Streptozotocin

Low doses of streptozotocin induced diabetes mellitus, combined with HFD led to steatosis, ballooning and inflammation in the liver, gradually progressed to fibrosis and an increased risk of liver cancer [98]. Furthermore, Lisa Lo et al. also used a combination study of HFD and streptozotocin and similarly found MASLD lesions and fibrosis [99].

### 4.2. Carbon Tetrachloride

Carbon tetrachloride (CCl_4_) is a widely used model to study liver fibrosis. However, CCl_4_ alone induces liver fibrosis without causing obesity or insulin resistance. Therefore, CCl_4_ is typically used in combination with HFD to achieve a more comprehensive model [100]. Kubota et al. administered HFD followed by multiple injections of CCl_4_ to mice. They found that mice fed HFD alone exhibited only fatty liver without features of steatohepatitis. In contrast, the CCl_4_ and HFD group showed significant steatosis, inflammatory lesions in liver lobules, ballooning, fibrosis, and markedly elevated serum transaminase levels [101]. However, the mean body weight of mice injected with CCl_4_ was significantly lower and glucose and cholesterol levels were lower than the control levels [102]. Subsequently, Liu et al. used the same modeling approach on a rat model and obtained similar pathological results [101].

### 4.3. Diethylnitrosamine

Diethylnitrosamine (DEN) is a chemical carcinogen that promotes cancer by affecting cell cycle regulatory proteins, thereby accelerating the progression of liver fibrosis to hepatocellular carcinoma [103]. Thompson et al. induced liver cancer in mice using HFD and DEN, resulting in a 60% cancer incidence and increased oxidative stress in the liver [104]. Furthermore, MCD was used in combination with DEN to induce liver cancer in mice; it was found that the combination of different MASLD diet models and DEN promoted DEN-induced carcinogenesis [105]. Although chemical-induced models can be established relatively quickly, they often exhibit severe drug toxicity, high mortality in experimental animals, and significant differences in pathogenesis and mechanisms compared to human MASLD.

## 5. Conclusions

To gain more precise understanding of the pathogenesis and mechanisms of MASLD, researchers have been seeking animals that are more appropriate models of human physiology and pathology. Although rodents reproduce quickly and are small, they are not genetically and physiologically homologous to humans, whereas non-human primates have certain advantages, but are expensive in contrast to mice, which are more widely used in the study of MASLD. MASLD induction models exhibit different characteristics. Genetic models have the advantage of mainly exhibiting the metabolic syndromes associated with MASLD and undergo a very similar lesion process. Although HFD-induced animal models closely resemble human MASLD in terms of pathophysiology and phenotype, they require longer modeling times and often do not exhibit the MASH phenotype. To overcome this limitation, a diet containing fructose, trans fats and cholesterol can be employed. In contrast, MCD and CDAA dietary models do not induce metabolic syndromes similar to those in humans and have distinct pathophysiological mechanisms. Therefore, these models are primarily suited for liver-specific research [76,81]. On the other hand, high fructose diets are often preferred by researchers and combined with other dietary models, more effectively replicate the progression of human diseases. Chemically induced models, such as those using CCl_4_ in combination with the MASLD diet, result in liver fibrosis that may not fully reflect the human disease. However, they are valuable for studying advanced fibrosis, liver cirrhosis, and liver cancer.

Currently, there is no animal model that fully replicates the pathophysiology of human MASLD. An ideal MASLD model should accurately reflect the histopathology and pathophysiology of the human liver while considering critical factors such as survival rates, modeling time, success rates, and reproducibility.
